# Role of Arginase 2 in Murine Retinopathy Associated with Western Diet-Induced Obesity

**DOI:** 10.3390/jcm9020317

**Published:** 2020-01-22

**Authors:** Reem T. Atawia, Katharine L. Bunch, Abdelrahman Y. Fouda, Tahira Lemtalsi, Wael Eldahshan, Zhimin Xu, Alan Saul, Khaled Elmasry, Mohamed Al-Shabrawey, Ruth B. Caldwell, R. William Caldwell

**Affiliations:** 1 Department of Pharmacology and Toxicology, Medical College of Georgia at Augusta University, Augusta, GA 30912, USA; ratawia@augusta.edu (R.T.A.); katharine.bunch@gmail.com (K.L.B.); weldahshan@augusta.edu (W.E.); 2 Vascular Biology Center, Medical College of Georgia at Augusta University, Augusta, GA 30912, USA; afouda@augusta.edu (A.Y.F.); tlemtalsi@augusta.edu (T.L.); zhxu@augusta.edu (Z.X.); rcaldwel@augusta.edu (R.B.C.); 3 Department of Ophthalmology, Medical College of Georgia at Augusta University, Augusta, GA 30912, USA; asaul@augusta.edu; 4 Vision Discovery Institute, Medical College of Georgia at Augusta University, Augusta, GA 30912, USA; malshabrawey@augusta.edu; 5 Department of Oral Biology, Dental College of Georgia at Augusta University, Augusta, GA 30912, USA; elmasryanatomy@gmail.com; 6 Department of Human Anatomy and Embryology, Faculty of Medicine, Mansoura University, 35516 Mansoura, Egypt; 7 Charlie Norwood VA Medical Center, Augusta, GA 30912, USA

**Keywords:** arginase 2, inflammasome, oxidative stress, obesity/diabetes-induced retinopathy

## Abstract

Western diet-induced obesity is linked to the development of metabolic dysfunctions, including type 2 diabetes and complications that include retinopathy, a leading cause of blindness. Aberrant activation of the inflammasome cascade leads to the progression of obesity-induced pathologies. Our lab showed the critical role of arginase 2 (A2), the mitochondrial isoform of this ureahydrolase, in obesity-induced metabolic dysfunction and inflammation. A2 deletion also has been shown to be protective against retinal inflammation in models of ischemic retinopathy and multiple sclerosis. We investigated the effect of A2 deletion on western diet-induced retinopathy. Wild-type mice fed a high-fat, high-sucrose western diet for 16 weeks exhibited elevated retinal expression of A2, markers of the inflammasome pathway, oxidative stress, and activation of microglia/macrophages. Western diet feeding induced exaggerated retinal light responses without affecting visual acuity or retinal morphology. These effects were reduced or absent in mice with global A2 deletion. Exposure of retinal endothelial cells to palmitate and high glucose, a mimic of the obese state, increased expression of A2 and inflammatory mediators and induced cell death. These effects, except for A2, were prevented by pretreatment with an arginase inhibitor. Collectively, our study demonstrated a substantial role of A2 in early manifestations of diabetic retinopathy.

## 1. Introduction

Obesity and type-2 diabetes (T2D) represent a severe health threat in developed nations worldwide. Furthermore, diabetes-induced retinal pathology and dysfunction is the major cause of impaired vision and blindness in working-age adults and therefore represents a significant economic burden [1,2]. Factors involved in the pathogenesis of diabetic retinopathy include inflammation, oxidative stress, and reduced endothelial nitric oxide (NO) production [3,4,5,6]. Studies have indicated that obesity and diabetes substantially affect the retina and its interactive vascular, neuronal, glial, and immune cells [5,7].

We, and others, previously reported the crucial role of arginase in the development of diabetes-induced pathologies and dysfunctions [3,8,9,10]. Arginase, a key enzyme in the urea cycle, can reciprocally regulate NO production by competing with nitric oxide synthase (NOS) for their shared substrate, L-arginine [11,12]. This ureohydrolase exists as two isoforms: Arginase 1, which is located in the cytoplasm and highly expressed in the liver, and arginase 2 (A2), which is primarily mitochondrial. Both isoforms are found in a variety of cells, including endothelial, smooth muscle, neuronal, immune, and retinal cells. Additionally, both can be upregulated under conditions of high glucose and increased reactive oxygen species (ROS) [13,14,15,16].

Our 2019 study in mice fed a high-fat, high-sucrose (HFHS) diet for 16 weeks showed that mice with a global A2 deletion (A2^−/−^) exhibited lower body weight gain, lower fasting blood glucose and insulin levels, improved metabolic and vascular endothelial function, and decreased circulating ROS levels compared to wild-type (WT) HFHS-fed mice [17]. WT mice on the HFHS diet displayed significantly higher body weight, as early as 9 weeks of age, when compared to both WT ND-fed mice and A2^−/−^ mice on the same HFHS diet. The HFHS-fed WT mice also exhibited elevated visceral adipose tissue (VAT) inflammation and fibrosis, pro-inflammatory macrophage VAT infiltration, systemic oxidative stress, and metabolic and peripheral vascular endothelial dysfunction. All these alterations were markedly reduced with A2 deletion [17].

Studies in a variety of mouse models have shown that deletion of A2 protects against retinal neurovascular injury and preserves retinal function with injury and in disease states, including ischemia-reperfusion injury, retinopathy of prematurity, multiple sclerosis, and optic neuropathy [18,19,20,21]. High fat diets have been shown to elicit a state of chronic inflammation as evidenced by increased activation of microglia and macrophage infiltration [4].

Obesity-induced retinal inflammation occurs particularly via nucleotide-binding oligomerization domain (NOD)-like receptor pyrin domain-containing protein 3 (NLRP3) inflammasome signaling, resulting in the activation of downstream effectors including caspase-1 and interleukin-1β (IL-1β), which are associated with ROS elevation. This is observed early during the progression of retinopathy in endothelial cells and macrophages and contributes to the late stage functional and structural deficits. Increased retinal ROS, which include peroxynitrite (ONOO^−^) and lipid peroxides, are observed in several models of diabetic retinopathy (DR) and contribute to inflammasome activation [4,5].

Several studies have suggested a link between western diet-induced obesity, the development of retinopathy, and retinal microvascular abnormalities. The mechanism underlying this association involves activation of inflammatory processes, increased production of ROS, and expression of inflammatory cytokines [4,5]. To investigate these, we assessed the levels of NLRP3 inflammasome signaling, peroxynitrite (ONOO^−^) and peroxidation of lipids, and IL-1β. To further assess the state of inflammation in the retina, immunofluorescent double labeling of Iba1 (a microglia/macrophage-specific calcium-binding protein) and the proinflammatory cytokine, interleukin-6 (IL-6), was performed on retina sections [22,23].

While the high fat diet mimics several aspects of obesity-associated retinopathy, the addition of sucrose in the HFHS murine diet more closely mirrors the modern western diet, producing similar pathophysiologic conditions to those observed in humans [24]. With this refined murine model of diet-induced obesity, we sought to investigate the potential benefit of global A2 deletion on western diet-induced retinopathy with regards to increased inflammation via activation of the NLRP3 inflammasome pathway and its effects on retinal histology and function.

## 2. Materials and Methods

### 2.1. Mouse Model of Obesity

All animal procedures were performed following the ARVO Statement for the Use of Animals in Ophthalmic and Vision Research and were approved by the Institutional Animal Care and Use Committee at Augusta University. Male, wild type (WT) mice and mice with a global deletion of arginase 2 (A2^−/−^) from a C57BL/6J background were used in this study. The development, breeding, and genotyping of these animals was previously described [17]. All animals were maintained at ambient temperature on a 12:12 h light/dark cycle and were fed ad libitum. Mice were fed either a normal chow diet (ND) (calorie percentage: 18% fat, 24% protein, 58% carbohydrate with approximately 5% from sucrose; Envigo (formerly Harlan), Huntingdon, Cambridgeshire, UK) or a western diet of high fat and high sucrose (HFHS) (calorie percentage: 59% fat, 15% protein, 26% carbohydrate with 20% from sucrose; F#1850, Bio-Serv, Flemington, NJ, USA). The diets were started four weeks after birth and continued for 16 weeks to mimic the metabolic syndrome milieu. Our 2019 publication involving the mice used in this present study provided their body weight over the course of the study and characterized their fasting blood glucose, serum insulin levels, and systemic metabolic functions [17].

### 2.2. Tissue Collection and Preparation

At 20 weeks of age, mice were anesthetized with a ketamine/xylazine cocktail injected intraperitoneally and euthanized by exsanguination. One whole eye and one retina were collected from each mouse. Isolated retinas were flash frozen and stored at −80 °C until homogenized in RIPA buffer with protease and phosphatase inhibitors for western blot analysis. Whole eyes were fixed in 4% paraformaldehyde (PFA) overnight at 4°C followed by 72-h cryopreservation in 30% (*w*/*v*) sucrose solution and embedded in optimal cutting temperature (O.C.T.) compound at −80 °C prior to sectioning for immunofluorescence studies [25].

### 2.3. Western Blot

Retina protein lysates were separated on sodium dodecyl sulfate (SDS)-polyacrylamide gels and transferred to nitrocellulose membranes where they were blocked in 5% non-fat milk (Bio-Rad, Hercules, CA, USA) and then incubated overnight at 4°C with one of the following primary antibodies prepared in 2% bovine serum albumin or in 5% non-fat milk. A2 (Santa Cruz Biotechnology Cat. No. Sc-20151, Dallas, TX, USA; 1:250), anti-3-Nitrotyrosine (Sigma-Aldrich Cat. No. N0409, St. Louis, MO, USA; 1:1000), NLRP3 (Cell Signaling Technology Cat. No. 15101, Danvers, MA, USA; 1:500), poly (ADP-ribose) polymerase (PARP) (Cell Signaling Technology Cat. No. 9542, Danvers, MA, USA; 1:500), caspase 1 (Cell Signaling Technology Cat. No. 2225, Danvers, MA, USA; 1:500), IL-1β (R & D Systems, Cat. No. AF-401-NA, Minneapolis, MN, USA; 1:1000), 4-hydroxynoneal (4HNE) (Abcam Cat. No. ab46545, Cambridge, MA, USA; 1:1000), Tubulin (Sigma-Aldrich Cat. No. T-9026, St. Louis, MO, USA; 1:5000), heat shock protein 90 (Hsp90) (BD Biosciences, Cat. No. 610418, Franklin Lakes, NJ, USA; 1:1000) and β-actin (Sigma-Aldrich Cat. No. A1978, St. Louis, MO, USA; 1:5000). The following day, membranes were washed three times in TBS-T (Tris-buffered saline with 0.5% Tween-20) and then incubated with the corresponding horseradish peroxidase-conjugated secondary antibody (GE Healthcare, Piscataway, NJ, USA; 1:2500) for one hour at room temperature. Signals were detected using an enhanced chemiluminescence system (GE Healthcare Bio-Science Corp., Piscataway, NJ, USA) and quantified by densitometry using ImageJ software (version 1.49, National Institutes of Health, Bethesda, MD, USA) and normalized to the loading control.

### 2.4. Immunofluorescence

Retina sections were soaked in 1× PBS for 5 min, blocked for 1 h (10% normal donkey serum in 1× TBS-T), and incubated overnight at 4 °C in blocking buffer with an anti-A2 antibody (Abcam Cat. No. ab228700, Cambridge, MA, USA; 1:250). Sections were washed three times for 10 min in 1× PBS and incubated in a secondary antibody conjugated to Oregon Green 488 (Molecular Probes Cat. No. O-6381, Eugene, OR, USA; 1:500) for one hour at 37 °C. Sections were washed three times for 10 min in 1× PBS and mounted with SlowFade Gold Antifade Reagent with DAPI (Life Technologies Cat. No. S36938). Imaging was performed using a Carl Zeiss 780 multiphoton laser scanning upright confocal microscope (Zeiss LSM780, Carl Zeiss Microscopy, White Plains, NY, USA).

All IL-6 labeling steps were performed on ice. First, frozen retina sections were blocked for 20 min (5% normal goat serum, 0.01% bovine serum albumin, 0.3% Triton-X in 10mM Hepes pH 8.2). The samples were subsequently blocked in an Avidin/Biotin Blocking Kit (Vector Laboratories Cat. No. SP-2001, Burlingame, CA, USA) for a total of 40 min, as per the manufacturer’s protocol. Then, samples were incubated in a biotinylated IL-6 antibody (BD BioSciences Cat. No. 554402, Franklin Lakes, NJ, USA; 1:200) for 1 h. Post-incubation, samples were washed three times in 1× PBS for five minutes and then incubated for 30 min in an Avidin-Texas Red conjugated secondary antibody (Vector Laboratories Cat. No. A-2006, Burlingame, CA, USA; 1:400). Samples were subsequently washed three times with 1× PBS and incubated overnight at 4 °C in the primary antibody, ionized calcium binding adapter molecule 1 (Iba1: A marker of macrophages and microglia) (Wako Laboratory Chemicals Cat. No. 019-19741, Neuss, Germany; 1:100). After three washes, the sections were incubated in secondary antibody conjugated to Oregon Green 488 (Molecular Probes Cat. No. O-6381, Eugene, OR, USA; 1:200) for one hour at 37 °C. The sections were washed three times in 1× PBS and mounted with Vectashield Antifade Mounting Medium (Vector Laboratories Cat. No. H-1000, Burlingame, CA, USA). Imaging was performed using a Carl Zeiss 780 multiphoton laser scanning inverted confocal microscope (Zeiss LSM780, Carl Zeiss Microscopy, White Plains, NY, USA).

### 2.5. Retinal Optical Coherence Tomography (OCT)

Optical coherence tomography (OCT) (Envisu OCT, Bioptigen, Inc., Morrisville, NC, USA) was performed on mice kept anesthetized with isoflurane/O_2_. Pupils of the anesthetized mice were dilated with 1% tropicamide ophthalmic drops prior to image acquisition. Lubricant eye gel (GenTeal; Novartis Pharmaceuticals, East Hanover, NJ, USA) was used throughout the procedure to maintain corneal moisture and clarity [26]. Morphometric analysis of retinal thickness was assessed using ImageJ software (version 1.49, National Institutes of Health, Bethesda, MD, USA).

### 2.6. Electroretinography (ERG)

To assess the effect of HFHS diet and A2 deletion on retinal function, dark-adapted (scotopic) and light-adapted (photopic) electroretinography (ERG) was performed. Following overnight dark adaptation, mice were anesthetized using isoflurane and prepared for ERG under dim red lighting. A rectal probe-linked heating pad was used to keep body temperature at 37 °C. Then, proparacaine, tropicamide, and phenylephrine drops were administered to each eye for dilation and topical anesthesia. Silver thread electrodes were placed on each eye and a drop of hypromellose was added to protect the cornea from drying. An optic fiber was then positioned in front of each pupil to deliver visual stimuli, ranging from about 0.018 to 14.4 candela-second/meter^2^ (cd-s/m^2^), generated by an LED device (Lightspeed Technologies, Campbell, CA, USA). Experiments were performed using a series of scotopic tests with 5 ms flashes of increasing luminance [18]. For the scotopic flashes, a-, b-, and c-waves were measured conventionally.

### 2.7. Visual Acuity Assessment

A virtual optokinetic system (OptoMotry, CerebralMechanics, Medicine Hat, AB, Canada) was used to assess visual acuity. Vertical sine-wave black and white gratings moving at 12°/s or a gray uniform field of the same mean luminance were projected on computer monitor screens as a virtual cylinder revolved around an unrestrained mouse standing on a pedestal at the epicenter. The gray field was projected while the mouse was moving on the pedestal, but when movement ceased, the gray field was replaced with the grating. Grating rotation under these circumstances elicited reflexive visual optokinetic tracking, which was scored by live video using a staircase procedure with a yes/no response, as spatial frequency increased. A spatial threshold was generated at 100% contrast through each eye separately but interleaved in the testing session [27].

### 2.8. Bovine Retinal Endothelial Cell Culture and the Effect of Obesity Conditions

Bovine retinal endothelial cells (BRECs) were isolated as previously described [28]. Cells from passages 5–9 were cultured in complete M199 medium (Gibco Thermo Fisher, Waltham, MA, USA) containing 10% fetal bovine serum (FBS), penicillin/streptomycin (Gemini, West Sacramento, CA, USA) until they reached 70–80% confluency. Cells were then grown in M199 medium containing 0.2% FBS, 0.1% bovine serum albumin (BSA), and 50 µM L-arginine [29]. Cells were exposed to media containing either normal glucose concentration (NG: 5 mM) or BSA or media containing high-glucose concentration (HG: 25 mM) and BSA-conjugated palmitate (PA: 100 μM) for 72 h. One group of cells was treated with the arginase inhibitor, 2-(S)-amino-6-boronohexanoic acid (ABH, 100 μM) for one hour before exposure to PA + HG treatment. Cells were then harvested for western blotting or fixed for cell death assessment determination using a terminal deoxynucleotidyl transferase dUTP nick end labeling (TUNEL) assay kit (Millipore, Billerica, MA, USA). DNase I was used as a positive control in some of the wells treated with BSA only. Positive control wells were pretreated with DNase buffer (30 mM Trizma base pH 7.2, 4 mM MgCl_2_, 0.1 mM DTT) at room temperature for five minutes. DNase I was applied with a final concentration of 0.5 μg/mL and incubated for 10 min at room temperature. Afterwards, wells were washed three times with distilled water. Following this, all wells were stained according to the manufacturer’s protocol, mounted with SlowFade Gold Antifade Reagent with DAPI (Life Technologies Cat. No. S36938, Carlsbad, CA, USA) and imaged at 20× on a Carl Zeiss 780 multiphoton laser scanning inverted confocal microscope (Zeiss LSM780, Carl Zeiss Microscopy, White Plains, NY, USA). Analysis of percentage of TUNEL positive cells was performed blindly using ImageJ software (Version 1.49, National Institutes of Health, Bethesda, MD, USA).

### 2.9. Statistical Analysis

Results are expressed as mean ± standard error of the mean (SEM). Statistical analyses were tested by GraphPad Prism 7 and 8 (GraphPad Software Inc., La Jolla, CA, USA) using the unpaired student’s two-tailed *t*-test, the one-way ANOVA, or two-way ANOVA, followed by Tukey’s post hoc tests as appropriate. For ERG studies, two-way ANOVAs were used to gauge the effects of the genotype across stimulus intensities, and the effects at individual intensities were computed by *t*-tests after Holm–Bonferroni correction for the multiple comparisons. Values of *p* < 0.05 were considered significant.

## 3. Results

### 3.1. High-Fat, High-Sucrose (HFHS) Diet Increased Retinal Expression of A2

**Figure 1:**
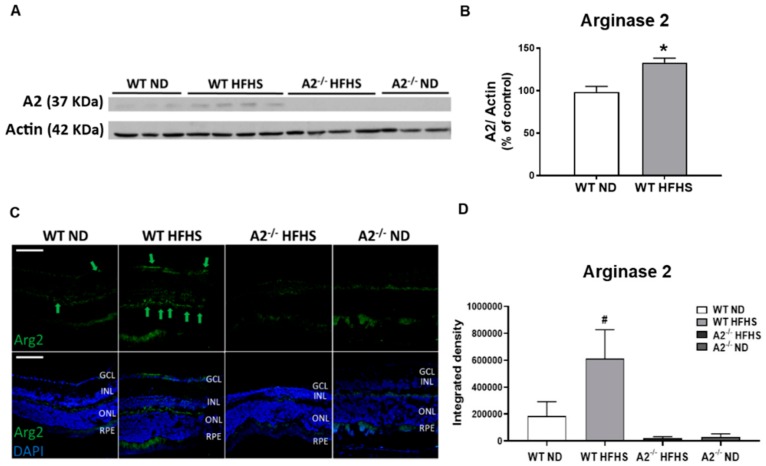
High-fat, high-sucrose (HFHS) diet increased retinal expression of arginase 2 (A2). Representative western blot with quantitation (**A**,**B**) showing elevated retina A2 protein levels in wild-type (WT) HFHS group and no A2 expression in arginase 2 knockout (A2^−/−^) animals (*n* = 5–6 per group). * *p* < 0.05 when compared to normal diet-fed (ND) mice within the same genotype. Representative images of immunofluorescent labeling of A2 (green) in retina cross-sections at 20× (**C**). Green arrows indicate A2 expression locations, scale bar = 50 µm (GCL: Ganglion cell layer, INL: Inner nuclear layer, ONL: Outer nuclear layer, RPE: Retinal pigment epithelium). Quantification of A2 expression in mouse retinas (*n* = 5 per group) (**D**), where # *p* < 0.05 when compared to animals with different genotypes on the same diet.

In our study, WT mice fed HFHS showed a significant increase in A2 protein expression as determined by western blot and immunofluorescence in retinal tissues compared to the WT ND group (Figure 1A–D, respectively). The A2^−/−^ mice showed no specific expression. The localization of prominent A2 immunofluorescence at the border of the inner and outer plexiform layers (Figure 1C) suggested that elevation of A2 was occurring in horizontal cells.

### 3.2. A2 Deletion Prevented HFHS Diet-Induced Oxidative Stress

**Figure 2:**
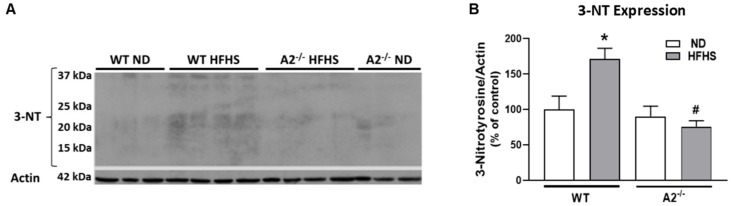

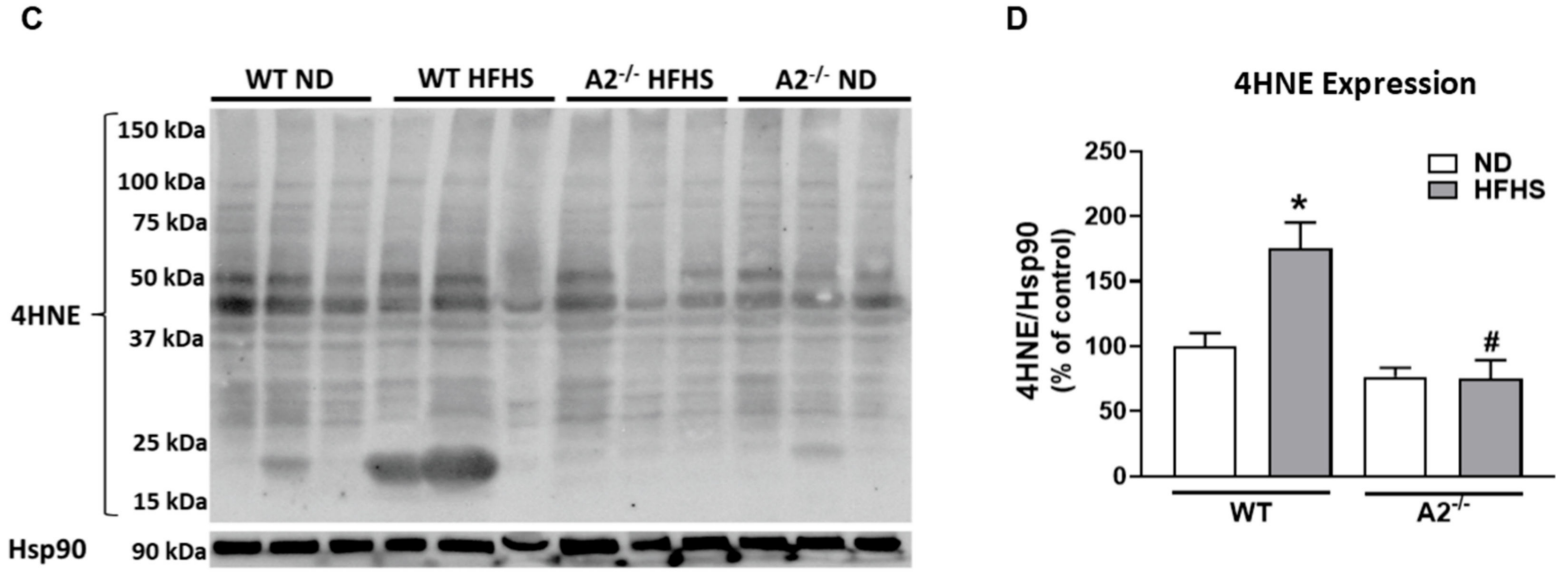
High-fat, high-sucrose (HFHS) diet increased retinal levels of tyrosine nitration and lipid peroxidation and this was prevented with arginase 2 (A2) deletion. Representative western blots and quantitation of 3-nitrotyrosine (3-NT) (*n* = 3–4 per group) (**A**,**B**) from same membrane as Figure 1A and 4-hydroxynoneal (4HNE) (*n* = 4 per group) (**C**,**D**) levels in retinas. Retinas from A2^−/−^ groups showed no elevation of 3-NT or 4HNE. Data are presented as mean ± SEM. * *p* < 0.05 compared to ND-fed mice of same genotype, # *p* < 0.05 compared to wild-type (WT) mice on same diet.

Peroxynitrite (ONOO^−^) is a reactive oxygen/nitrogen species that nitrates protein tyrosine moieties to produce 3-nitrotyrosine (3-NT). Levels of ONOO^−^ can be assessed indirectly by western blot analysis of 3-NT [30]. Lipid peroxidation, a form of oxidative stress, produces 4HNE, which covalently binds molecules containing amino groups. Western blot analysis showed that retinal levels of both 3-NT and 4HNE were elevated in WT HFHS mice compared to those on the normal diet (Figure 2A–D). Deletion of A2 prevented the HFHS diet-induced increase in these oxidative stress markers (Figure 2B,D).

### 3.3. A2 Deletion Prevented HFHS-Induced Retinal Inflammation and Inflammasome Activation

**Figure 3:**
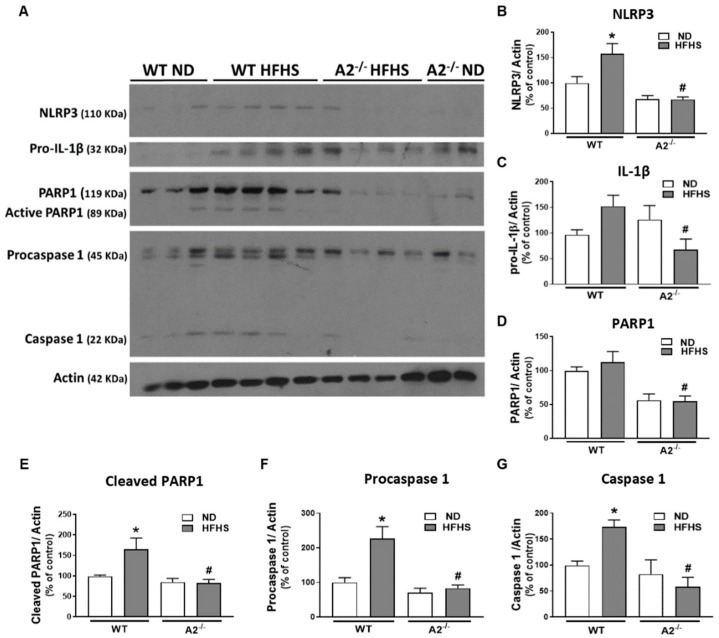
Arginase 2 (A2) deletion prevented high-fat, high-sucrose (HFHS) diet-induced inflammasome activation. Representative western blot (**A**) with quantitation showing the effect of HFHS and A2 deletion on the expression of nucleotide-binding oligomerization domain (NOD)-like receptor pyrin domain-containing protein 3 (NLRP3)) (**B**), pro-interleukin-1β (pro-IL-1β) (**C**), poly [ADP-ribose] polymerase 1 (PARP1) (**D**), active (cleaved) PARP1 (**E**), procaspase 1 (**F**) and caspase 1 (**G**) (*n* = 5–6 per group). Data are presented as mean ± SEM (standard error of the mean). * *p* < 0.05 when compared to normal diet (ND)-fed mice of the same genotype, # *p* < 0.05 when compared to wild-type (WT) mice on the same diet.

Western blot analysis confirmed significant increases in measures of retinal inflammation and inflammasome activation in WT mice on the HFHS diet. Levels of NLRP3, active (cleaved) PARP, procaspase 1, and caspase 1 were increased (Figure 3). In contrast, HFHS fed mice lacking A2 showed significant reductions in NLRP3 expression (Figure 3A,B) as well as its downstream effectors: procaspase 1, caspase 1, active (cleaved) PARP, and pro-IL-1β, compared to WT mice (Figure 3A–G). Our results indicate that A2 is involved in retinal inflammatory responses induced by a western-style diet.

**Figure 4:**
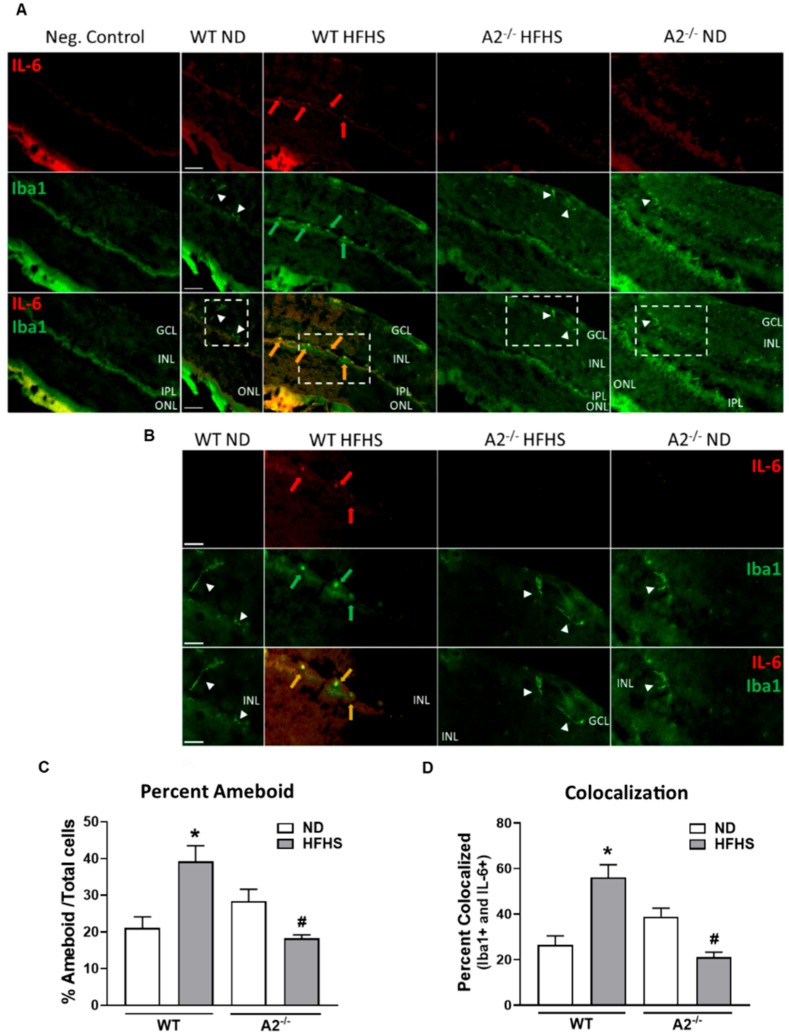
Arginase 2 (A2) deletion prevented high-fat, high-sucrose (HFHS)-induced retinal microglia activation/pro-inflammatory macrophage infiltration. Representative images at 20× (**A**) and 40× (**B**) showing immunofluorescent double-labeling of the inflammatory marker, interleukin-6 (IL-6) (red) and microglia/macrophage marker, ionized calcium binding adaptor molecule 1 (Iba1) (green), in retina sections of wild-type (WT) and A2^−/−^ mice fed a normal diet (ND) or HFHS diet (*n* = 5 per group). Boxes with dashed lines on 20× images illustrate the same section imaged at 40× shown below (**B**). Red arrows point to regions of IL-6 expression, green arrows point to ameboid microglia/macrophages, and orange arrows point to co-localization of IL-6 and Iba1 (yellow) in ameboid microglia or infiltrating inflammatory macrophages. White arrow heads point to ramified resident microglia. GCL: Ganglion cell layer, IPL: Inner plexiform layer, INL: Inner nuclear layer, ONL: Outer nuclear layer. Scale bars = 50 μm on WT ND images. ImageJ quantification of the number of activated ameboid microglia/macrophages compared to the total number of Iba1 positive cells (**C**), and the percentage of Iba1 positive cells expressing IL-6 (colocalization) compared to the total number Iba1 positive cells (**D**). Five randomly selected images per animal were quantified. * *p* < 0.05 when compared to ND-fed mice within the same genotype, # *p* < 0.05 when compared to WT on the same diet.

Additionally, we determined the effects of the HFHS diet and A2 deletion on retinal inflammatory immune cell responses. HFHS diet fed WT mice exhibited an 80% increase in number of activated (ameboid) microglia/ macrophages (Iba1 positive cells) compared to those on the normal diet (Figure 4A–C). Deletion of A2 prevented the HFHS-induced activation of microglia/infiltration of macrophages (Figure 4A–C). Similarly, A2^−/−^ mice on the HFHS diet did not exhibit an increase in Iba1 positive cells expressing the inflammatory cytokine, IL-6 (Figure 4A,B,D).

### 3.4. No Effect of HFHS Diet or A2 Deletion on Retinal Morphology

**Figure 5:**
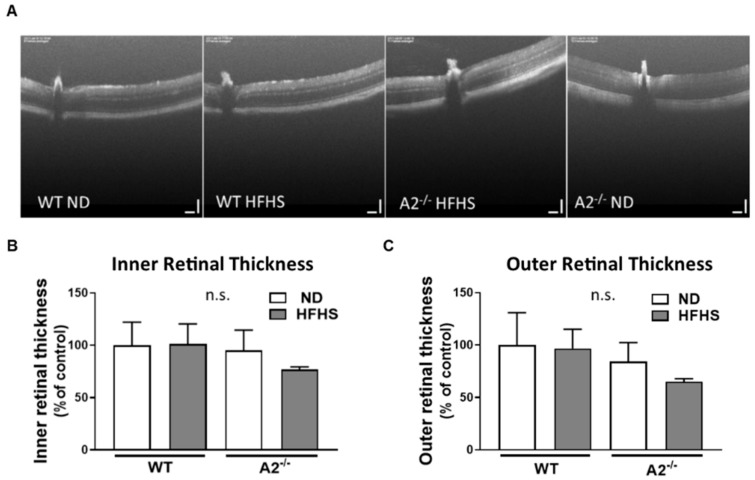
High-fat, high-sucrose (HFHS) diet and arginase 2 (A2) deletion had no effect on gross retinal morphology. Representative images of optical coherence tomography (OCT) in live mice (**A**) with quantitation of inner (**B**) and outer retinal thickness (**C**). n.s. = not significant. Data are presented as mean ± SEM (standard error of the mean) (*n* = 3 per group).

To determine the effect of the HFHS diet and A2^−/−^ on gross retinal morphology and to detect retinal cell loss, the thickness of the inner and outer retinal layers was assessed using optical coherence tomography (OCT). No differences were detected in the thickness of either the inner or outer retinal layers among all the four experimental groups (Figure 5A–C).

### 3.5. A2 Deletion Limited Abnormal HFHS Diet-Induced ERG Responses Without Affecting Visual Acuity

**Figure 6:**
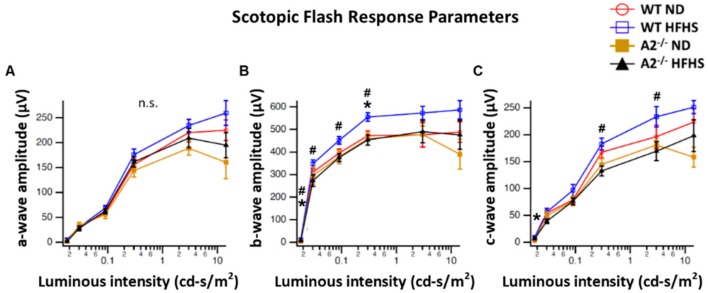

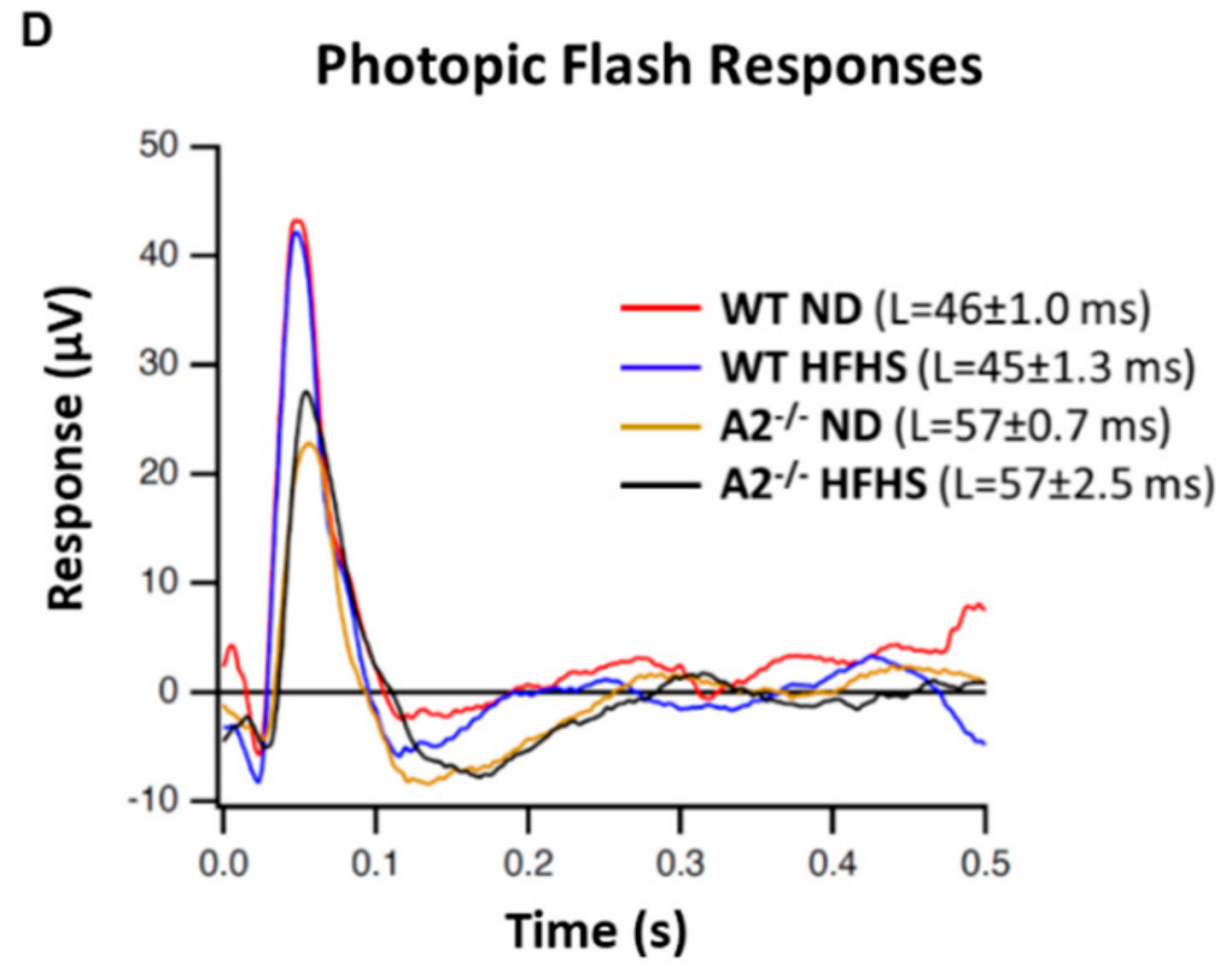
Electroretinogram (ERG) responses were recorded after overnight dark adaptation from mice in scotopic conditions and amplitudes were analyzed as a function of light intensity (*n* = 5 per group). Scotopic flash responses are provided for the a-, b-, and c-wave ERG components (**A**–**C**, respectively). Mean amplitudes with standard errors are plotted against luminance for each group. Averaged photopic responses (**D**) evoked by brief (5 ms) flashes are shown for each group. * *p* < 0.05 for the difference between the WT HFHS and WT ND groups, # *p* < 0.05 for the difference between the WT HFHS and A2^−/−^ HFHS groups.

Neurological deficits in the retina and abnormal response to light are considered important components of diabetic retinopathy progression [31,32,33]. In this study, a-wave amplitude was similar among all four groups (Figure 6A). However, WT mice fed the HFHS diet exhibited a significant increase in b-wave amplitude compared to the WT ND group (Figure 6B). A2^−/−^ mice fed HFHS showed responses similar to those seen in the ND-fed mice. Amplitudes of c-waves differed significantly at some intensities (Figure 6C). No significant differences were seen in latencies of the a-wave, b-wave and c-wave. Photopic response amplitudes were reduced for brief flashes in the A2^−/−^ mice. Latencies were also significantly longer for the A2^−/−^ groups (Figure 6D).

**Figure 7:**
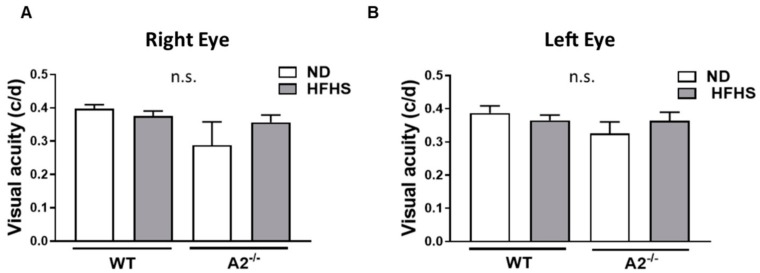
High-fat, high-sucrose (HFHS) diet and arginase 2 (A2) deletion did not affect visual acuity. Visual acuity threshold was measured as the optokinetic tracking response and recorded as cycles/degree (c/d) in wild-type (WT) and A2^−/−^ mice fed the normal diet (ND) or HFHS diet for 16 weeks (**A**,**B**) (*n* = 5–6 per group) n.s. = not significant.

The effect of HFHS diet-induced obesity on visual acuity was also assessed via optokinetic tracking (OKT) responses. No significant differences were observed among the four groups of mice (Figure 7A,B).

### 3.6. Inhibition of Arginase Blocked Inflammasome Activation and Apoptosis in Bovine Retinal Endothelial Cells (BRECs) Treated with Palmitate and High Glucose

**Figure 8:**
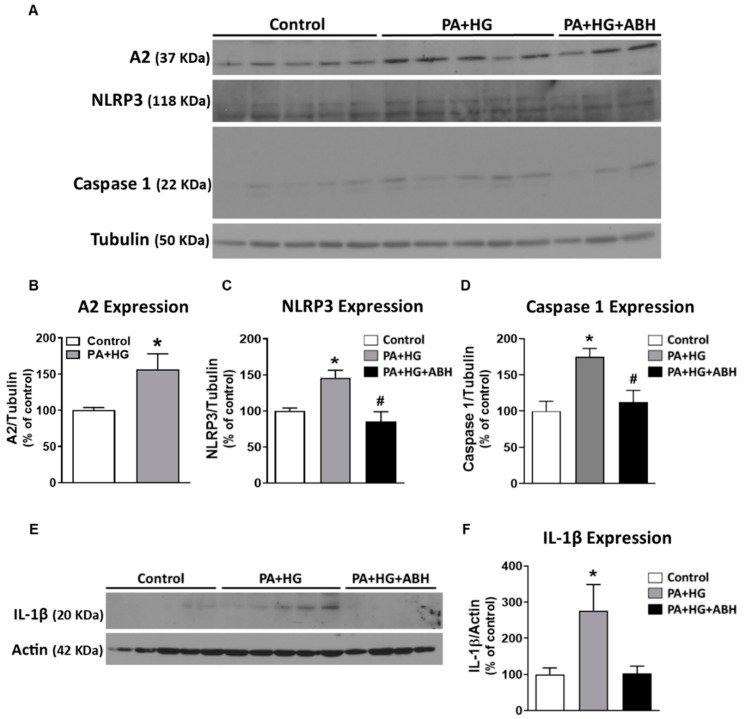
Inhibition of arginase limited inflammasome activation in bovine retinal endothelial cells (BRECs) treated with palmitate and high glucose (PA+HG). Representative western blots (**A**) of BRECs treated for 72 h with 100 µM palmitate and 25 mM of glucose (PA+HG) in the presence or absence of 2(S)-amino-6-boronohexanoic acid (ABH) showing the expression of A2 (**B**) and components of the inflammasome: nucleotide-binding oligomerization domain (NOD)-like receptor pyrin domain-containing protein 3 (NLRP3) (**C**), caspase 1 (**D**) and pro-interleukin-1 β (pro-IL-1β) (**E**,**F**) (*n* = 4–6 per group). Data are presented as mean ± SEM (standard error of the mean). * *p* < 0.05 compared to control (bovine serum albumin (BSA)-treated) group, # *p* < 0.05 compared to PA+HG treated group.

In order to test the direct effects of the in vivo conditions created by the HFHS diet on retinal endothelial cells, BRECs were exposed to 100 µM palmitate and 25 mM glucose (PA+HG) for 72 h. The PA+HG treatment induced a significant elevation of A2 protein expression (Figure 8A,B). These conditions also increased expression of NLRP3 inflammasome signaling moieties, NLRP3, caspase 1, and pro-IL-1β, compared to BSA (vehicle) treatment (Figure 8A,C–F). These effects on inflammasome components were blocked or blunted by pretreatment of cells with the arginase inhibitor, ABH.

**Figure 9:**
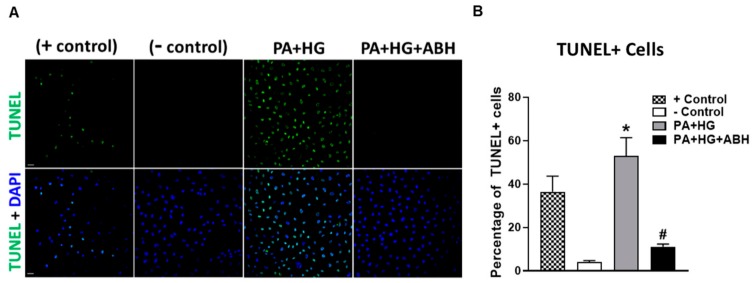
Representative terminal deoxynucleotidyl transferase dUTP nick end labeling (TUNEL) photomicrographs at 20× with quantitation (**A**,**B**) showing increased percentage of TUNEL positive cells in bovine retinal endothelial cells (BRECs) treated for 72 h with PA+HG (scale bar = 100 µm, *n* = 5–7 per group). Cell nuclei are labeled with DAPI (blue) and TUNEL positive cells fluoresced green. Data are presented as mean ± SEM (standard error of the mean). * *p* < 0.05 compared to control (BSA-treated) group, # *p* < 0.05 compared to PA+HG-treated group. Scale bar = 50 μm.

Additionally, we examined the effects of PA+HG on BREC apoptosis using a terminal deoxynucleotidyl transferase dUTP nick-end labeling (TUNEL) kit. DNase I was used as a positive control. Compared to BSA-treated cells, PA+HG treated cells showed a significant increase in TUNEL positive cells, which was blocked by pretreatment with ABH (Figure 9A,B).

## 4. Discussion

Obesity and type 2 diabetes are associated with increased retinal microvascular abnormalities primarily due to increased inflammation, oxidative stress, and endothelial dysfunction [34,35,36,37]. Diabetic retinopathy (DR) is the leading cause of blindness worldwide and is commonly associated with both type 1 and type 2 diabetes mellitus, the latter of which, is a common consequence of obesity [1,38]. Currently, anti-vascular endothelial growth factor (VEGF) intravitreal injections, steroidal agents, and laser ablation surgeries are the only available DR therapies. Though they have some moderate effectiveness, they have high potential for serious side effects and are often cost prohibitive [39,40,41].

Thus, understanding the underlying pathogenesis of the disease and investigating new potential targets for DR treatment is crucial. The most common experimental model to study DR utilizes the pancreatic β-cell toxin, streptozotocin (STZ) [42]. However, this model primarily mimics human type 1 diabetes and has limitations, including off-target effects [4,43]. The *db/db* genetic mouse model with a leptin-receptor mutation was used to mimic human type 2 diabetes (T2D) experimentally, however, leptin signaling itself affected the retina [44]. The western diet, characterized by high fat and high sucrose, induced obesity, insulin resistance, and diabetes in rodents and was characterized by the slow onset of type 2 diabetic complications [24]. This included retinopathy, which closely mirrors human pathophysiology without loss of inner retinal thickness. The disease progression with the high-fat, high-sucrose (HFHS) diet was mild and mimics early human retinopathy [24].

Previous studies have shown the involvement of A2 in several retinal pathologies including ischemia-reperfusion injury, retinopathy of prematurity, and retinal injury following optic nerve trauma [18,19,21]. In this study, retinas from WT mice fed the HFHS diet for 16 weeks showed a significant increase in A2 expression. Use of mice with a global deletion of A2 (A2^−/−^), allowed us to assess the role of A2 in the pathogenesis of obesity-induced retinopathy.

Chronic inflammation, which is associated with increased expression of pro-inflammatory cytokines and chemokines, was involved in the pathogenesis of DR and results in early micro-angiopathies such as capillary permeability, edema, inflammatory cell infiltration, tissue destruction, and neovascularization [45]. A recent study showed that mice fed a high fat diet developed initial retinal inflammation, followed by neurological deficits and subsequent retinal vascular abnormalities and permeability. The chronic inflammatory state involved activation of the NLRP3-inflammasome pathway in retinal endothelial cells and macrophages early in high fat diet-induced diabetic retinopathy [4,5]. Inflammasome pathway activation can further lead to pyroptosis, a caspase-dependent, inflammasome-mediated, subset of cell death [46]. Caspase 1 and, to some extent, caspase 7, are both activated by the inflammasome complex, and induce the cleavage of the DNA damage sensor, poly (adenosine 5′-diphosphate-ribose) polymerase 1 (PARP1). Cleaved PARP1 mediated the process of apoptosis by acting as a dominant negative fragment to inhibit PARP1-mediated DNA repair [47]. Similar results were observed in our model; retinas from WT mice fed the HFHS diet showed increased retina microglia/macrophage activation and increased expression of NLRP3-inflammasome components. Deletion of A2 prevented immune cell activation and blocked HFHS diet-induced increase in expression of NLRP3, IL-1β (pro-inflammatory cytokine), caspase 1, and cleaved PARP1. In support of these findings, A2 deletion was shown to dampen retinal inflammatory responses and inflammasome activation following optic nerve injury [21]. Also, our lab and others demonstrated the protective effect of A2 deletion against HFHS-induced inflammatory macrophage infiltration in adipose [17] and hepatic tissue [48].

Obesity and diabetes are associated with increased ROS production, which is believed to be the link to the pathogenesis of metabolic related complications [49]. Increased ROS levels activate the NLRP3 inflammasome pathway and increase production of inflammatory cytokines which, in-turn, increase ROS production [50]. Previous studies showed that high fat diets induced retinal oxidative stress, which supports our results [5]. Our lab also showed that A2 deletion can effectively ameliorate HFHS diet-induced increases in plasma lipid peroxides [17]. Additionally, A2^−/−^ mice were protected against retinal oxidative stress induced by ischemia-reperfusion injury and hyperoxia [18,25]. It has been suggested that enhanced polyamine metabolism, a pathway downstream of arginase, as evidenced by increased spermine oxidation, contributes to increased retinal oxidative stress [25]. Future studies are needed to determine the mechanism of A2-induced retinal oxidative stress and the effects of A2 deletion on antioxidant capacities.

Additionally, we were interested in the effect of HFHS-diet induced obesity/diabetes and A2 deletion on retinal function. Although diabetes induced by rapid depletion of insulin with use of streptozotocin promotes thinning of the inner retina as early as 2 months after diabetes induction, no apparent changes in retinal thickness were observed in animals fed high fat diet up to 12 months [4]. This agreed with our findings and with clinical studies showing no thinning of the inner nuclear retinal layer in the early stages of diabetes [51,52].

To evaluate retinal function after 16 weeks on the HFHS diet and to assess the effect of A2 deletion, electroretinography (ERG) and visual acuity tests were performed. In the current study, WT, but not A2^−/−^ mice, fed the HFHS diet showed abnormally elevated b-wave responses to light. This could be attributed to their hyperglycemic state, which is characterized by increased intracellular levels of Ca2^+^ and cGMP, that may increase response amplitudes and sensitivity to light stimuli [53]. Other studies have shown that scotopic responses are lower in models of high fat diet-induced obesity [54,55]. Another study showed impaired electroretinogram (ERG) responses after 6 months of high fat diet consumption [4]. In clinical settings, diabetic patients can present with subnormal, supernormal as well as normal electroretinogram (ERG) responses compared to healthy subjects [56,57].

To assess the effect of diet and A2 deletion on visual activity in conscious, freely moving animals, we used a virtual optokinetic system. We found no changes in visual acuity in our model, which was supported by a study using Ins2Akita mice, which showed visual performance deficits at 5 months but not at 2.5 months of age. This indicated an age or duration-dependent effect of diabetes on visual function [58].

Our in vivo data suggest that A2 has an important role in activating and perpetuating inflammatory processes such as NLRP3 inflammasome activation the early stages of DR. Obesity-induced inflammation is associated with high plasma levels of saturated non-esterified fatty acids (NEFA), particularly palmitate, which has been shown to activate retinal endothelial inflammasome signaling [5,59]. To model HFHS diet-induced obesity *in vitro*, bovine retinal endothelial cells (BRECs) were treated with PA+HG for 72 h, which resulted in induced expression of inflammasome components and increased cell death. These effects were attenuated by inhibition of arginase.

## 5. Conclusions

Collectively, our data showed that A2 was critically involved in HFHS diet-induced retinal oxidative stress, inflammation, microglia/macrophage activation, and exaggerated ERG light responses. Further studies are needed to investigate the effect of A2 deletion on retinal complications resulting from long-term HFHS consumption.
